# Review of Image-Processing-Based Technology for Structural Health Monitoring of Civil Infrastructures

**DOI:** 10.3390/jimaging10040093

**Published:** 2024-04-16

**Authors:** Ji-Woo Kim, Hee-Wook Choi, Sung-Keun Kim, Wongi S. Na

**Affiliations:** Department of Civil Engineering, Seoul National University of Science and Technology, Seoul 01811, Republic of Korea; gukim97@seoultech.ac.kr (J.-W.K.); cheesew01@seoultech.ac.kr (H.-W.C.); cem@seoultech.ac.kr (S.-K.K.)

**Keywords:** image processing, damage type, structural health monitoring, artificial intelligence

## Abstract

The continuous monitoring of civil infrastructures is crucial for ensuring public safety and extending the lifespan of structures. In recent years, image-processing-based technologies have emerged as powerful tools for the structural health monitoring (SHM) of civil infrastructures. This review provides a comprehensive overview of the advancements, applications, and challenges associated with image processing in the field of SHM. The discussion encompasses various imaging techniques such as satellite imagery, Light Detection and Ranging (LiDAR), optical cameras, and other non-destructive testing methods. Key topics include the use of image processing for damage detection, crack identification, deformation monitoring, and overall structural assessment. This review explores the integration of artificial intelligence and machine learning techniques with image processing for enhanced automation and accuracy in SHM. By consolidating the current state of image-processing-based technology for SHM, this review aims to show the full potential of image-based approaches for researchers, engineers, and professionals involved in civil engineering, SHM, image processing, and related fields.

## 1. Introduction

For structural health monitoring (SHM), the integration of image-processing techniques marks a significant evolution in how we perceive, assess, and maintain structural integrity. SHM is vital for ensuring the safety and longevity of infrastructure, which has traditionally relied on up-to-date sensor-based technology where finding and repairing damage at an early stage could extend the lifespan of a structure [1,2]. Furthermore, the incorporation of advanced image processing into SHM presents a transformative shift, offering a nuanced and comprehensive approach to structural-condition assessment. These image-processing techniques leverage visual data obtained through a variety of imaging methods, including drones equipped with cameras and thermal and ultrasonic imaging [3,4,5]. They enable a detailed examination of structural damages, deformations, and alterations that might elude traditional sensor-based monitoring. This visual narrative not only identifies but also visualizes the extent and nature of structural changes, facilitating proactive maintenance strategies. The significance of integrating image-processing techniques into SHM lies in addressing inherent challenges encountered in conventional monitoring approaches. These techniques surpass the limitations of conventional sensors, particularly in detecting subtle or localized damage that might otherwise remain unnoticed. Moreover, they provide a visual context, enhancing our understanding of structural deformations and damages, thereby aiding in informed decision making regarding maintenance and repair strategies.

Regarding SHM, image-processing techniques encompass a diverse array of methodologies. These methodologies encompass various stages of analysis, including edge detection, texture analysis, and image registration [6,7,8]. Edge detection involves identifying boundaries within an image and highlighting structural features and discontinuities that may indicate potential damage or changes. Texture analysis focuses on characterizing the spatial arrangement of pixels, enabling the identification of patterns or anomalies in the structure’s surface. Image registration aligns and overlays multiple images of the same structure, allowing for precise comparisons and the detection of changes over time. Together, these techniques contribute to the comprehensive analysis of structural images in SHM, aiding in the early detection and monitoring of potential defects or structural deterioration. The utilization of the aforementioned techniques in SHM offers several distinct advantages. Firstly, they enable non-destructive evaluations, allowing for continuous monitoring without compromising the structural integrity of the system. Secondly, their detailed analysis of structural conditions empowers informed decision making concerning maintenance and repair strategies. The applications of these techniques span across industries, ranging from civil engineering to aerospace and mechanical systems, underscoring their universal relevance in safeguarding critical infrastructure.

The integration of image-processing techniques signifies a transformative phase in SHM, revolutionizing our ability to visualize, interpret, and respond to structural data. Its universal applicability across industries emphasizes its potential in ensuring the safety and durability of vital infrastructure. In this work, the authors have overviewed SHM, with a particular emphasis on the stages of image acquisition, image-processing techniques, and the integration of artificial intelligence (AI) for damage detection. This review of image acquisition elucidates the importance of capturing high-quality and relevant data for subsequent analysis. Subsequently, an in-depth examination of image-processing techniques highlights the pivotal role of edge detection, texture analysis, and image registration in enhancing the interpretability of structural images. Furthermore, the incorporation of AI in the damage-detection stage signifies a paradigm shift towards automated and efficient evaluation, offering a promising avenue for the real-time and accurate identification of structural anomalies.

## 2. Damage Types in Structural Health Monitoring

**Figure 1:**
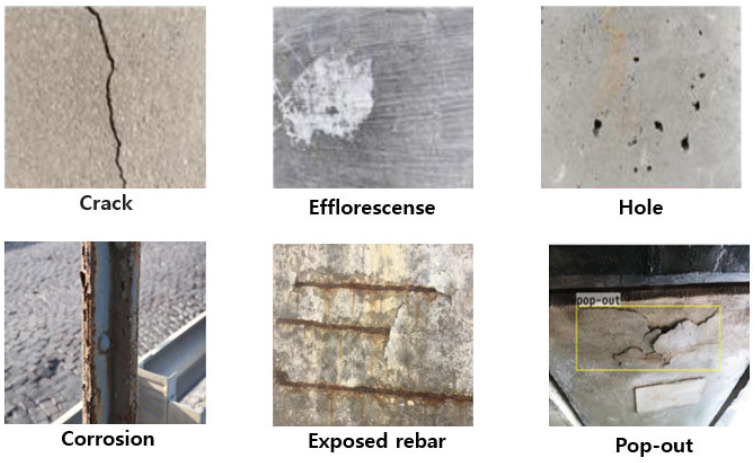
Different types of damages for structures [44,45,46,47].Reprinted with permission from ref. [44]. Copyright 2019 John Wiley and Sons, Adapted form [45], Reprinted with permission from ref. [46]. Copyright 2023 Elsevier, Reprinted with permission from ref. [47]. Copyright 2019 John Wiley and Sons.

A wide array of damages can affect structures (Figure 1), necessitating specific monitoring methods for their detection and evaluation. One of the most prevalent types of damage is the emergence of cracks within structures [9]. These cracks can arise from various sources, including excessive loading, material fatigue, or harsh environmental conditions. Detecting these cracks often involves employing a combination of visual inspections [10], acoustic emissions [11], ultrasonic testing [12], or utilizing sensors that measure strain or deformation [13,14,15,16,17,18]. Similarly, corrosion poses a significant threat to structural integrity, gradually deteriorating materials due to environmental reactions. Effective monitoring against corrosion encompasses diverse methods such as the use of electrochemical sensors [19], corrosion-potential measurements [20], visual inspections [21], and other non-destructive testing techniques [22,23,24].

Another critical concern is fatigue damage, resulting from cyclic stress on materials, leading to cumulative microstructural damage and eventual failure [25,26]. Techniques for monitoring fatigue include the deployment of strain gauges [27], vibration analysis [28,29], and the ongoing assessment of changes in material properties over time [30,31]. Impact events can also inflict damage, causing localized structural issues. Detecting such impact damage involves various methodologies such as acoustic-emission methods [32,33,34,35] and employing non-destructive testing techniques like ultrasound [36] or thermography [37]. Deformation, encompassing changes in the structure’s shape or size beyond its elastic limits, requires vigilant monitoring. To detect and track deformations, sensors like strain sensors [38], displacement sensors [39], and monitoring systems capable of tracking changes in structural geometry are employed [40]. Furthermore, delamination, wear and erosion, material degradation, and foundation and settlement issues, as well as water intrusion, each present distinct challenges to structural integrity.

However, while image-processing techniques play a crucial role in detecting and assessing various types of damage in SHM, it is essential to acknowledge that not all damage types can be effectively addressed solely through image processing. Some forms of structural deterioration, such as internal material degradation, complex subsurface defects, or issues requiring specialized sensing methods, may fall beyond the scope of traditional image-processing applications. Certain damage types, like material fatigue or internal corrosion within the structure, may necessitate more specialized testing methods such as advanced sensing technologies, laboratory analyses, or non-optical methods like acoustic monitoring. Internal defects that are not visually apparent or confined to subsurface layers may require techniques like ultrasonic techniques [41] and Ground-Penetrating Radar (GPR) [42,43], which provide insights beyond the capabilities of traditional image processing.

## 3. Image-Acquiring Method for SHM

### 3.1. Drone Equipped with Camera

**Figure 2:**
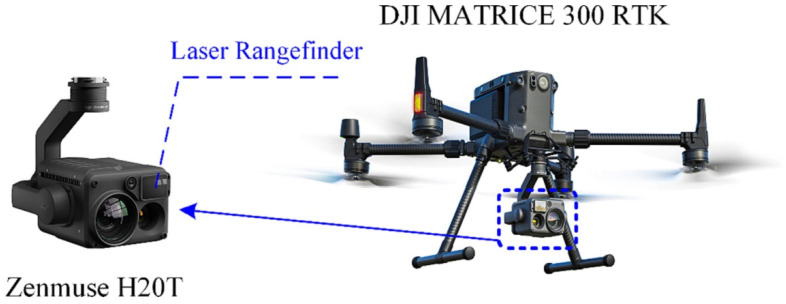
Components of UAV. Reprinted with permission from ref. [50]. Copyright 2023 Elsevier.

Drones equipped with high-resolution cameras have revolutionized various fields, including structural inspections and monitoring. These unmanned aerial vehicles (UAVs) offer a versatile and efficient means of capturing detailed imagery of structures that might otherwise be challenging to access. By leveraging these cameras mounted on drones, engineers and researchers can perform comprehensive SHM tasks. The aerial perspective provided by drones allows for the quick and thorough inspection of large areas, such as bridges, towers, or pipelines, offering detailed visual data for analysis. This approach not only enhances the safety of inspectors by reducing the need for risky physical access but also enables the collection of extensive and precise visual information, empowering better decision making for maintenance and ensuring the structural integrity of critical infrastructure. Research on using UAVs equipped with cameras can be found from various authors. Zhao et al. introduced a UAV-based 3D reconstruction model for dam emergency monitoring and inspection [48]. The structure-from-motion method (which is a photogrammetric range-imaging technique to estimate 3D structures using 2D image sequences that may be coupled with local motion signals) was adopted to create a high-precision 3D dam model. The results showed that the reconstruction model demonstrated satisfactory accuracy and substantially improved dam monitoring and inspection efficiency. Reagan et al. combined UAVs with a 3D digital image-correlation technique to perform non-contact, optically based measurements to monitor the health of bridges, where Zemuse H20T was installed onto the UAV for research, as shown in Figure 2 [49]. Results from the study show that the proposed method was able to detect changes to the bridge’s geometry with an uncertainty on the order of 10^−5^ m. Ding et al. proposed a UAV-based system for precise concrete-crack-detection and quantification without reference markers to quantify the cracks with widths less than 0.2 mm [50]. More research on using UAVs for acquiring images can be found by various authors [51,52,53,54,55,56].

### 3.2. Thermography

**Figure 3:**
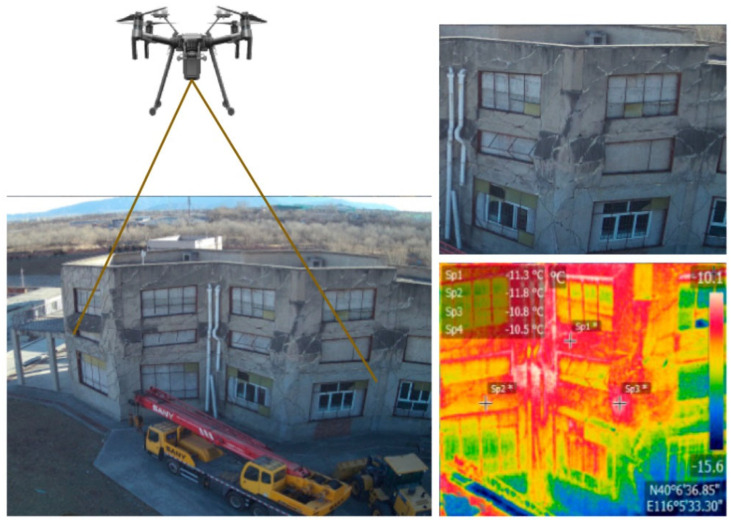
Infrared thermal detection of wall cracks of earthquake-damaged buildings. Adapted from [58].

Thermography is a useful method for acquiring images in SHM which serves as a non-destructive and non-contact method, providing valuable information for the real-time assessment of the health and integrity of structures. This technique involves capturing and analyzing the infrared radiation emitted by the surface of an object. In the context of SHM, thermography helps identify temperature variations in structures, offering insights into potential issues such as delaminations, voids, or moisture ingress. Thermal imaging cameras, commonly used in thermography for SHM, detect infrared radiation and produce images known as thermograms or thermographic images. These images depict the temperature distribution on the surface of the monitored structure. Irregularities in temperature patterns can indicate areas of concern, prompting further investigation or maintenance. Hellstein and Szawedo integrated infrared cameras with 3D scanners to create 3D thermography to enable the creation of 3D thermograms where the temperatures were mapped [57]. The proposed concept was tested during the diagnosis of several industrial composite structures including boats, planes, and wind turbine blades. Zhang et al. established a remote sensing information-extraction method that combined UAVs and infrared thermal imaging technology to automatically detect the structural damage of buildings [58]. The proposed research work was verified using the earthquake search-and-rescue simulation as an example to show an accuracy of 78%. Here, the concept of thermography on the wall cracks of earthquake-damaged buildings can be seen in Figure 3, where a thermographic image can be seen on the right side of the figure. Thermography can also be used to identify damages in bridges, as Omar and Nehdi explored the potential application for detecting subsurface delamination in concrete bridge decks [59]. The findings reveal that a UAV with high-resolution thermal infrared imagery offers an efficient tool for precisely detecting subsurface anomalies in bridge decks. As proven by various researchers, thermography is one of the vital technologies for acquiring required images for SHM [60,61,62,63].

### 3.3. Light Detection and Ranging (LiDAR) Technology

LiDAR (Light Detection and Ranging) technology stands as a significant asset to SHM by employing laser light to measure distances and create detailed three-dimensional representations of structures. The principle of LiDAR operation involves emitting laser pulses towards a target surface, measuring the reflected light’s time of return, and generating accurate distance measurements. This process results in the creation of precise point clouds that effectively represent the external geometry of the structure. It excels in monitoring structural deformations in real-time, capturing dynamic responses such as vibrations, and identifying surface defects like cracks. LiDAR boasts several advantages, including high precision in measurements and a non-intrusive nature of data collection, eliminating the need for physical contact with the structure. This makes LiDAR suitable for monitoring various structures without causing any disruption. However, LiDAR has limitations to consider. Its capabilities are primarily surface-focused, and it may not capture details beneath surfaces. Additionally, weather conditions and visibility can influence the effectiveness of LiDAR data acquisition.

**Figure 4:**
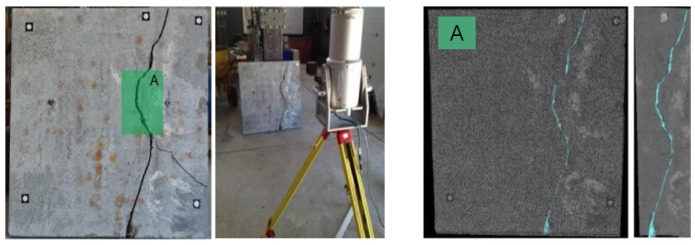
Concrete specimen laser scanning (**left**) and result values (**right**). A—represents the zoomed-in section of the Figure Reprinted with permission from ref. [69]. Copyright 2017 Elsevier.

UAV equipped with a lidar scanner can be automated for damage detection. One of these research findings can be found in the work of Yan et al. [64], where the authors customized a UAV platform with a high-resolution camera and a Velodyne VLP-16 lidar scanner to scan a bridge’s substructure, validating the proposed method’s effectiveness in recognizing concrete cracks with 85% accuracy and quantifying them with less than 10% error compared to manual annotations and measurements. More work on automated damage detection, and one of the most researched target structures, bridges, can be found in various works [65,66,67,68,69,70,71,72,73]; an example of using lasers for crack detection can be seen in Figure 4 [69].

### 3.4. Ultrasonic Imaging

**Figure 5:**
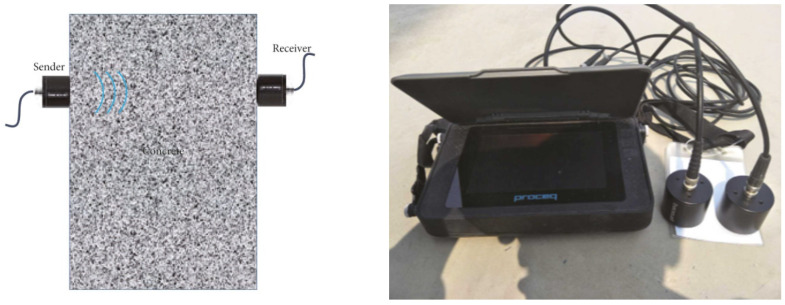
Ultrasonic concept on concrete (**left**) with the Pundit PL-200 sonicator (**right**). Adapted from [82].

Ultrasonic imaging, a non-destructive testing technique, plays a crucial role in SHM by utilizing high-frequency sound waves to penetrate materials and generate detailed images of the internal structure. The fundamental principle involves sending ultrasonic waves into a material, measuring the reflected waves’ time, and producing images that highlight structural features and potential anomalies. In SHM applications, ultrasonic imaging is instrumental in detecting anomalies like cracks, voids, and delamination within structures [74,75,76,77,78,79,80,81,82]. Figure 5 shows the general concept of ultrasonic testing for concrete, involving the transmission of ultrasonic waves from a sender (transducer) through the concrete, the detection of reflected waves by a receiver (transducer), and the analysis of the received signals to identify and characterize the defects within the material [82]. It can provide valuable insights into material properties, aiding in the characterization of structural elements, and facilitates thickness measurement for assessing structural integrity.

The image-acquisition process involves deploying ultrasonic transducers on the structure’s surface or embedding them within. These transducers emit ultrasonic waves, and the reflections from interfaces, anomalies, or defects are recorded and processed to create visual representations of the internal structure. Ultrasonic imaging offers several advantages, including high-resolution images for detailed structural analysis and real-time monitoring capabilities that enable timely interventions and maintenance. However, it also has limitations, such as a limited penetration depth in certain materials and the requirement for accessibility to both sides of the material being inspected.

### 3.5. Ground-Penetrating Radar

Ground-Penetrating Radar (GPR) is a non-destructive imaging technique used to assess subsurface conditions and identify anomalies within structures. The fundamental principle involves emitting short pulses of electromagnetic waves into the structure or ground and recording the reflections that return from the subsurface materials. This process generates detailed images or profiles of the subsurface, offering a cross-sectional view of the internal structure. GPR serves various crucial applications. Firstly, it excels in the detection of anomalies, such as cracks, voids, or delamination within structures. Secondly, it aids in characterizing materials by assessing properties like changes in moisture content, which can significantly impact structural integrity. Moreover, GPR provides depth profiling, offering valuable information about the depth at which anomalies or structural features are located.

**Figure 6:**
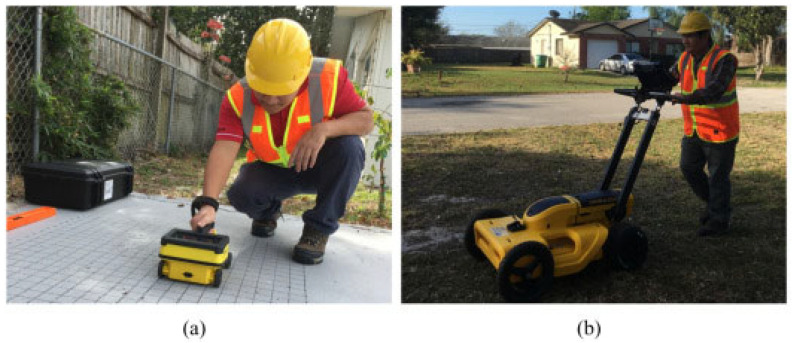
Using GPR for (**a**) concrete scanning and (**b**) utility locating. Reprinted with permission from ref. [83] Copyright 2021 Elsevier.

The image-acquisition process involves data collection, where GPR equipment transmits and receives electromagnetic pulses, recording the time taken for reflected signals to return; the general use can be seen in Figure 6 [83]. Subsequently, data-processing techniques are applied to create a subsurface profile, and skilled interpretation is crucial to identifying anomalies or structural characteristics. The processed data enables the real-time monitoring of subsurface conditions, facilitating timely interventions and maintenance strategies. GPR offers several advantages in SHM, including its non-destructive nature, allowing for assessments without causing harm to structures. The real-time monitoring capabilities contribute to proactive maintenance and structural-integrity management. However, GPR does have limitations, including depth constraints influenced by material properties and antenna frequency, as well as the need for skilled interpretation due to the complexity of the acquired images. To break these limitations, various authors have tackled the problem to enhance the technology [83,84,85,86,87,88,89,90].

### 3.6. Satellite Technology

Satellite technology employs various sensors to capture data related to structures and the surrounding environment. One key method is optical imagery, where satellites capture visible and infrared light reflected from the Earth’s surface. Optical sensors measure this reflected light, offering visual information about structures and environmental features. This method is widely used for visual assessment, monitoring changes over time, and identifying structural issues, applicable in diverse SHM contexts, including large-scale infrastructure and environmental monitoring [91,92,93].

Synthetic Aperture Radar (SAR) is another significant satellite-based technique. SAR utilizes microwave signals to penetrate cloud cover, detecting subtle deformations in structures and monitoring ground displacement. SAR is particularly valuable for monitoring large areas and regions with frequent cloud cover, making it applicable to diverse SHM applications [94,95,96,97]. In addition, Interferometric Synthetic Aperture Radar (InSAR) is an advanced technique analyzing interference patterns from multiple SAR images. InSAR is highly sensitive, capable of detecting millimeter-scale deformations in structures and monitoring ground subsidence. Its sensitivity makes it valuable for monitoring subtle changes in structures and landscapes, especially in applications such as infrastructure-stability assessment [98,99,100].

**Figure 7:**
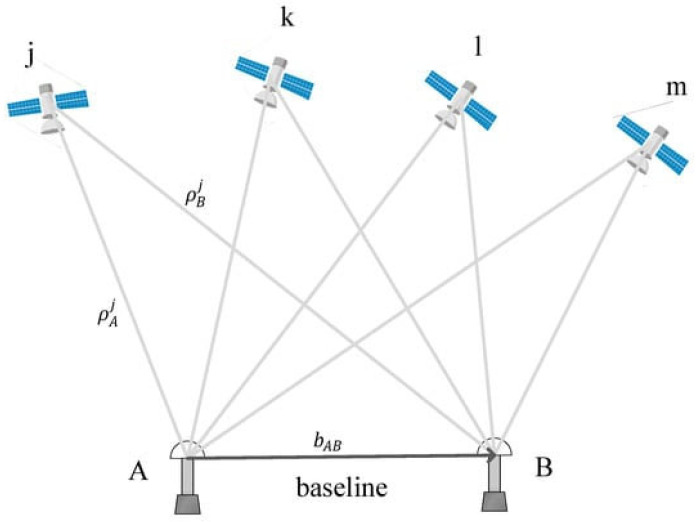
Basic concept of relative positioning. In RTK, the coordinates of the target point are determined by calculating the baseline vector between the target point and the reference point. Adapted from [93].

Satellite technology finds applications in SHM for large-scale monitoring, environmental impact assessment, and emergency response. Its advantages include wide coverage, enabling the monitoring of extensive infrastructures, remote sensing capabilities, and the ability to perform temporal analysis by capturing changes and trends in structural conditions over time. However, satellite imagery may have limitations such as lower spatial resolution compared to other imaging techniques, limiting the detection of small-scale details. Additionally, cost considerations and data accessibility, influenced by factors like cloud cover, may impact the availability of high-resolution satellite imagery. In summary, satellite technology is integral to SHM, providing a macroscopic view of structures and landscapes through optical, SAR, and InSAR techniques, with applications ranging from large-scale monitoring to environmental impact assessment and emergency response. (Figure 7).

## 4. Image-Processing Techniques for SHM

Image-processing techniques are crucial for extracting meaningful information from raw image data, enabling engineers and analysts to detect, analyze, and interpret structural health conditions accurately. Image processing involves techniques and methods used to manipulate, enhance, or analyze images. This includes tasks like noise reduction, edge detection, contrast enhancement, and segmentation, as image quality is often degraded due to factors including lighting conditions such as sunny or cloudy skies. In the context of damage identification for SHM, image-processing techniques are employed to pre-process images, enhance their quality, and extract relevant features or regions that might indicate damage or structural anomalies. For instance, edge detection might highlight cracks or structural discontinuities, while segmentation could isolate damaged areas for further analysis.

### 4.1. Edge Detection

**Figure 8:**
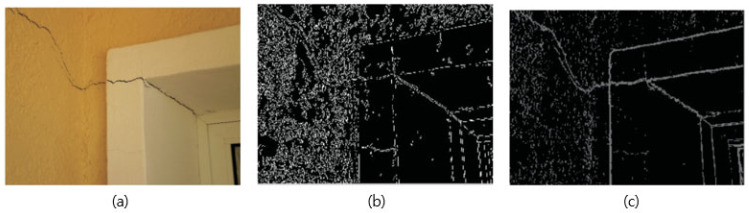
(**a**) Test image, (**b**) canny and (**c**) hyperbolic edge detectors for crack detection. Adapted from [100].

Edge detection is a computer-vision technique that can be employed as part of the image-processing component in SHM to identify discontinuities or changes in the structure which may indicate damage. Edge-detection algorithms aim to identify boundaries or edges within an image. In the context of SHM, these edges may represent cracks, fractures, or other structural anomalies. One of the latest review works on using image-processing techniques for crack analysis in structures was conducted by Azouz et al. [98]. From their research, the authors reviewed image-processing algorithms to identify the crack and analyze its properties to detect occurred damages. Here, several preprocessing approaches were employed to predict crack propagation, which included image enhancement and image filtering to remove the noise and blur. An example of image processing can be seen in Figure 8, showing typical corner cracks observed in masonry walls around door openings [101]. The cracks are detected through canny and hyperbolic tangent filters, and one can see that it can identify cracks along with other edges and corners. It is a common technique for image processing, on which more work can be found by various researchers [101,102,103,104,105,106,107,108,109].

### 4.2. Texture Analysis

Texture analysis involves a sophisticated exploration of spatial variations in pixel intensities across structural images where various authors have used it to detect damage including bridges and concrete structures [110,111,112,113,114,115,116]. This nuanced approach employs a repertoire of computational methods to delve into the intricate details of surface textures. Gabor filters, for instance, are adept at capturing both fine and coarse textures by analyzing local spatial frequency contents. The Gray Level Co-occurrence Matrix (GLCM), on the other hand, offers insights into the statistical relationships among pixel values, providing a measure of homogeneity, contrast, and entropy [117,118]. Wavelet transforms enable a multi-scale analysis, unraveling textures at different frequency bands. The aim of these techniques is to discern unique patterns associated with materials, finishes, or potential damage within the structural components. This goes beyond conventional image processing by adding a layer of semantic understanding to the visual data. The quantitative features derived from texture analysis serve as essential inputs for subsequent machine learning algorithms, facilitating the automated detection and classification of structural conditions. By meticulously interpreting textural nuances, this approach enhances the precision of anomaly detection, making it a cornerstone in the proactive monitoring and assessment of structural health over time.

### 4.3. Image Registration

Image registration is a process that aligns two or more images, taken at different times or from different perspectives, to ensure that corresponding features in the images are spatially aligned [119,120,121,122,123]. This alignment is crucial for various applications, including remote sensing, medical imaging, computer vision, and structural health monitoring. The primary goal of image registration is to correct geometric distortions and differences in scale, rotation, and translation, allowing for accurate the comparison and analysis of the images. The process involves identifying corresponding points or features in the images and applying spatial transformations to bring them into alignment. This alignment can be achieved through various algorithms and mathematical techniques. Furthermore, this alignment is crucial for tracking changes, deformations, or damages in the structure over time. While image registration itself is not a direct method for crack detection, it facilitates the comparison of images that may reveal structural changes, including the presence of cracks. Thus, one can say that this method is particularly valuable when dealing with dynamic structures or monitoring structural changes over extended periods.

**Figure 9:**
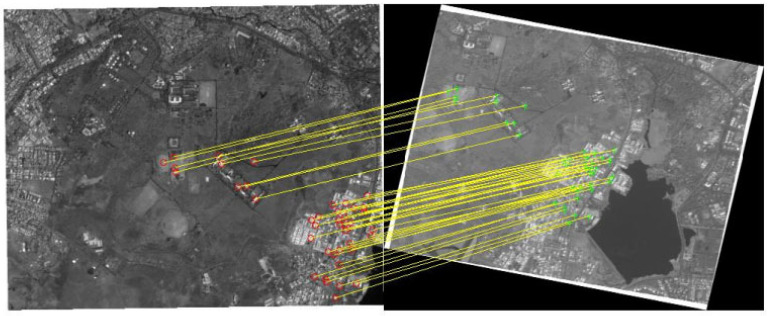
Inlier points using the RANSAC algorithm. The detected points (red circles) and matched point pairs (green circles) in both reference and slave image using SURF feature detectors are shown. Adapted from [124].

One common method for image registration in SHM is intensity-based registration [124,125,126]. This method often employs metrics like normalized cross-correlation or mutual information to align images based on pixel intensities. Normalized cross-correlation is particularly useful when the images have similar modalities, while mutual information is robust for cases where images are acquired using different modalities, such as combining visible and infrared imaging. In [124], the authors researched a hybrid approach given to an image-registration process that included the Random Sample Consensus (RANSAC) algorithm for the accurate assessment of remote sensing satellite images for image registration. The detected points and matched point pairs in both reference and slave image using SURF feature detectors are shown in Figure 9.

Another image-registration method is known as feature-based registration, which relies on identifying and matching distinctive features or key points in the images [127,128,129]. Point-based registration using algorithms like Scale-Invariant Feature Transform (SIFT) or Speeded-Up Robust Features (SURF) is common. These methods enable the identification of corresponding points between images, allowing for accurate alignment even in the presence of rotations or scale changes. Optical flow methods, such as the Lucas–Kanade method [130,131,132], estimate motion vectors between images, making them valuable for monitoring dynamic structural changes or vibrations. Geometric transformation methods, including B-spline registration or Thin-Plate Spline (TPS), offer flexibility for deformable registration, accommodating complex transformations.

### 4.4. Segmentation

Segmentation is a pivotal image-processing technique aimed at partitioning images of structures into distinct and meaningful regions. The primary objective is to identify and isolate specific structural components or features within the images, facilitating a detailed analysis of their conditions. Segmentation plays a crucial role in tasks such as crack detection, deformation monitoring, and material characterization, contributing to a comprehensive understanding of the structural health of monitored assets.

One common method employed for segmentation in SHM is thresholding [133,134,135,136]. This approach involves setting a threshold value to distinguish different regions based on pixel intensities. Global thresholding applies a uniform threshold across the entire image, while adaptive thresholding adjusts the threshold locally, enhancing adaptability to varying image conditions. Thresholding is particularly useful for applications where structural components exhibit distinct intensity differences, enabling the separation of different regions of interest. Another widely used method is edge-based segmentation, with the Canny Edge Detector being a prominent technique [137,138,139,140]. By identifying gradients and edges within an image, this method allows for the extraction of boundaries and the segmentation of regions based on changes in intensity. Edge-based segmentation is valuable for tasks like crack detection, where the presence of sharp intensity variations signifies potential structural issues.

**Figure 10:**
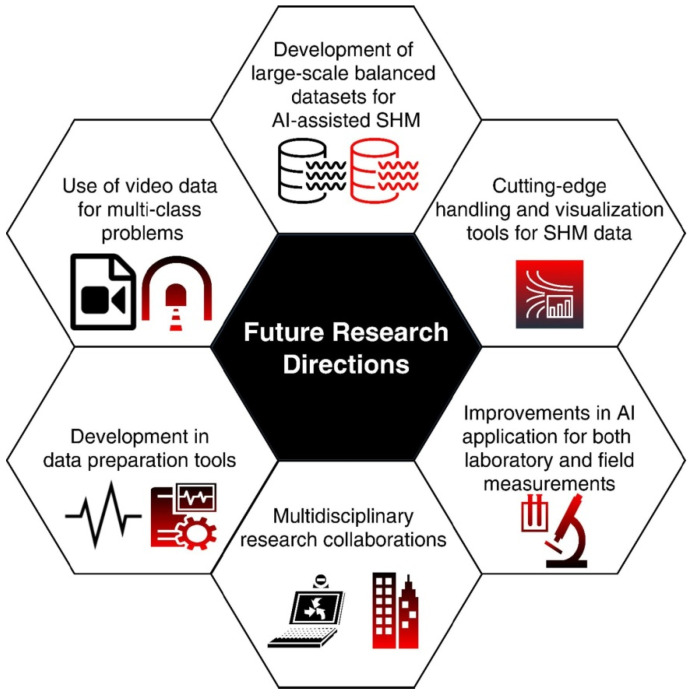
A schematic of the potential future research directions of CNN-based SHM research. Reprinted with permission from ref. [149]. Copyright 2021 Elsevier.

Region growing is a method that entails the iterative expansion of regions from seed points based on similarity criteria [141,142]. This technique is beneficial when analyzing homogeneous regions with similar properties, contributing to accurate segmentation for tasks such as material characterization. Watershed segmentation is an approach inspired by hydrology, treating pixel intensities as elevations [143,144]. It simulates flooding to separate regions, making it suitable for delineating complex structural features. Furthermore, clustering methods, such as K-Means Clustering, involve grouping pixels into clusters based on their similarity in feature spaces [145,146,147]. This technique is versatile, applicable to various SHM tasks, and particularly useful when structural components exhibit different characteristics. Finally, the advent of deep learning has introduced convolutional neural networks (CNNs) for segmentation tasks in SHM [148,149,150,151]. These models, trained on large datasets, automatically learn hierarchical features, proving effective for complex segmentation tasks. A good review work on CNN-based SHM can be found in the work by Sony et al., whose potential future research is summarized in Figure 10 [149].

## 5. Artificial Intelligence for the Post-Processing of Image Data

Artificial intelligence (AI) techniques are applied to post-processing in SHM through training models on large datasets that contain labeled examples of various structural conditions [152,153,154]. The trained models learn patterns and relationships between features, enabling them to make informed decisions during post-processing. In the case of deep learning, neural networks automatically learn hierarchical representations of data, allowing for complex feature extraction and interpretation. These AI techniques enhance the automation, accuracy, and efficiency of the post-processing stage, contributing to a more advanced and sophisticated analysis of structural health.

### 5.1. Machine Learning

Machine learning-based Classification in SHM involves leveraging algorithms to automatically categorize the health status of structures based on extracted features from sensor data or images. Among the common methods used for SHM, Support Vector Machines (SVM) stand out for their ability to find optimal hyperplanes for binary or multiclass classifications, making them effective in distinguishing between healthy and damaged states. Random Forest, an ensemble learning technique, is employed to handle large datasets and enhance classification robustness, and is particularly useful in the complex and dynamic environment of SHM [155,156,157,158,159,160,161]. 

### 5.2. Pattern Recognition

Unlike machine learning, which represents specific methodologies within the broader scope of pattern recognition, the concept itself is not tied to particular algorithms. Instead, it encompasses a diverse array of techniques, including classical statistical methods, rule-based systems, and various learning-based approaches. The primary objective of pattern recognition is to enable systems to recognize and make sense of regularities or structures present in data, facilitating informed decision making based on observed patterns.

In SHM, pattern recognition plays a pivotal role in deciphering anomalies or deviations in structural behavior that may signify damage or deterioration. Common techniques for pattern recognition in SHM include Principal Component Analysis (PCA) for dimensionality reduction [162], cluster analysis to group similar patterns [163], Hidden Markov Models (HMM) for modeling sequential data [164], and Autoencoders for unsupervised feature learning [165,166]. Other methods such as Self-Organizing Maps (SOM) [167], Support Vector Machines (SVM) [168,169], and ensemble techniques like bagging and boosting also contribute to pattern recognition in SHM [170,171], aiding in the automatic identification of structural anomalies and providing valuable insights for maintenance and decision making. The choice of a specific method depends on factors such as the nature of the data, the complexity of structural conditions, and the requirements of the SHM application.

## 6. Current Limitations and Challenges in Image-Based SHM

In the domain of structural health monitoring (SHM), leveraging image-based techniques presents several challenges that influence the effectiveness and reliability of monitoring systems. One significant challenge lies in the complex interpretation of captured images, requiring the precise identification and categorization of various structural anomalies. Distinguishing between normal variations, environmental effects, and actual structural issues demands advanced image-analysis techniques and expert interpretation. Furthermore, the quality and resolution of captured images significantly influence the accuracy of damage detection and characterization. Limitations in image quality due to environmental factors, distance from the target, or equipment constraints might hinder the precise analysis and interpretation of structural conditions. The sheer volume of data generated by high-resolution images poses a practical challenge, necessitating efficient storage, transmission, and processing capabilities. Handling large datasets requires robust algorithms and computing resources to ensure timely and accurate analysis.

The real-time processing and monitoring of structures using image-based techniques remain demanding. Achieving rapid processing and the interpretation of images to detect emerging damage or changes in structural conditions is essential for proactive maintenance, yet it presents ongoing challenges in terms of computational speed and accuracy. The standardization and validation of image-based SHM techniques are essential for ensuring consistency and reliability across different monitoring systems. Establishing standardized protocols and benchmarks while validating these techniques against established methods remains a critical step for their widespread adoption and trust within the industry. Environmental variability poses a significant challenge in image-based SHM. Variations in lighting, weather conditions, or obstructions can impact image quality and consistency, making it challenging to ensure reliable and consistent monitoring.

Integrating image-based SHM with other sensor-based monitoring techniques for a comprehensive assessment of structural health requires synchronization, data fusion, and the creation of unified analysis frameworks. Achieving seamless integration while maintaining accuracy and reliability across different monitoring systems is a complex endeavor. The automation of image analysis using machine learning algorithms for SHM requires robust training datasets, sophisticated algorithms, and continuous improvement efforts to ensure high accuracy and reliability. Developing automated systems that can effectively process and interpret image data in real-time remains a significant challenge. The accessibility and deployment of imaging systems in various locations, especially in remote or inaccessible areas, present logistical challenges. Ensuring the accessibility, ease of installation, and reliability of imaging systems across diverse environments remains a crucial consideration.

Finally, the cost implications associated with implementing high-quality imaging equipment and advanced image-processing systems can be substantial. Striking a balance between costs and the effectiveness of image-based SHM is vital for its widespread adoption and practicality in real-world applications. Addressing these multifaceted challenges involves continual advancements in imaging technology, algorithm development, standardization efforts, and interdisciplinary collaboration. Overcoming these obstacles will enhance the capabilities and reliability of image-based SHM, ensuring the safety and longevity of critical infrastructure.

## 7. Conclusions

In conclusion, this review paper provides an overview of image-processing-based technologies for the structural health monitoring (SHM) of civil infrastructures. Through the exploration of various damage types that exist for structures, the discussion on image-acquisition methods for SHM, image-processing techniques utilized after acquiring the images, and the emerging role of artificial intelligence in post-processing image data, key insights have been reviewed in this work.

While image-processing techniques are pivotal for identifying and evaluating different types of damage within SHM, not all forms of structural deterioration can be found solely through image processing. Certain types of damage, like internal material degradation, intricate subsurface defects, or issues necessitating specialized sensing methods, may surpass the capabilities of conventional image-processing applications. Thus, the selection of appropriate image acquisition methods, whether using traditional cameras or advanced sensors like LiDAR, infrared or ultrasonic, significantly influences the quality and reliability of SHM data. Furthermore, the diverse spectrum of image-processing techniques, ranging from traditional filters to advanced algorithms like convolutional neural networks, offers a rich toolkit for analyzing and interpreting structural data accurately and efficiently. Recommendations include exploring hybrid approaches combining different techniques to maximize information extraction and improve detection accuracy.

In practical applications, image-processing-based SHM technologies find widespread use in various civil-engineering projects. From monitoring the structural integrity of bridges and tunnels to assessing the health of buildings and pipelines, these technologies play a pivotal role in ensuring the safety, reliability, and longevity of critical infrastructures.

## Data Availability

Data are contained within the article.

## References

[1] Farrar C.R., Worden K. (2007). An introduction to structural health monitoring. Philos. Trans. R. Soc. A.

[2] Domaneschi M., Martinelli L., Cucuzza R., Noori M., Marano G.C. (2023). Structural Control and Health Monitoring Contributions to Service-life Extension of Bridges. ce/papers.

[3] Pham G.N., Nguyen P.H. (2020). Drone Detection Experiment Based on Image Processing and Machine Learning. Int. J. Sci. Res..

[4] Chrysafi A.P., Athanasopoulos N., Siakavellas N.J. (2017). Damage detection on composite materials with active thermography and digital image processing. Int. J. Therm. Sci..

[5] Ciampa F., Pickering S.G., Scarselli G., Meo M. (2017). Nonlinear imaging of damage in composite structures using sparse ultrasonic sensor arrays. Struct. Control Health Monit..

[6] Shrivakshan G.T., Chandrasekar C. (2012). A comparison of various edge detection techniques used in image processing. Int. J. Comput. Sci. Issues.

[7] Öztürk Ş., Akdemir B. (2015). Comparison of edge detection algorithms for texture analysis on glass production. Procedia Soc. Behav. Sci..

[8] Kong X., Li J. (2018). Image registration-based bolt loosening detection of steel joints. Sensors.

[9] Mohan A., Poobal S. (2018). Crack detection using image processing: A critical review and analysis. Alex. Eng. J..

[10] Yeum C.M., Dyke S.J. (2015). Vision-based automated crack detection for bridge inspection. Comput.-Aided Civ. Infrastruct. Eng..

[11] Moradian Z., Einstein H.H., Ballivy G. (2015). Detection of Cracking Levels in Brittle Rocks by Parametric Analysis of the Acoustic Emission Signals. Rock Mech. Rock Eng..

[12] Yang H., Yang L., Yang Z., Shan Y., Gu H., Ma J., Wu Z., Tian T., Ma S., Wu Z. (2023). Ultrasonic detection methods for mechanical characterization and damage diagnosis of advanced composite materials: A review. Compos. Struct..

[13] Laflamme S., Cao L., Chatzi E., Ubertini F. (2016). Damage detection and localization from dense network of strain sensors. Shock. Vib..

[14] Yan Y., Hao H., Yam L. (2004). Vibration-based construction and extraction of structural damage feature index. Int. J. Solids Struct..

[15] Benedetti M., Fontanari V., Zonta D. (2011). Structural health monitoring of wind towers: Remote damage detection using strain sensors. Smart Mater. Struct..

[16] Kwon Y., Park C., Kim J., Kim H., Park C., Lee B., Jeong Y., Cho S.J. (2020). Effects of bending strain and crack direction on crack-based strain sensors. Smart Mater. Struct..

[17] Wang P.F., Toshiyuki T., Takanori T., Hiroyuki M. (2013). Early fatigue damage detecting sensors—A review and prospects. Sens. Actuators A.

[18] Chen Z., Lin X.Q., Yan Y.H., Xiao F., Khan M.T., Zhang S. (2020). Noncontact group-delay-based sensor for metal deformation and crack detection. IEEE Trans. Ind. Electron..

[19] Figueira R. (2017). Electrochemical Sensors for Monitoring the Corrosion Conditions of Reinforced Concrete Structures: A Review. Appl. Sci..

[20] Parthiban T., Ravi R., Parthiban G.T. (2006). Potential monitoring system for corrosion of steel in concrete. Adv. Eng. Softw..

[21] Stewart M.G. (2010). Reliability safety assessment of corroding reinforced concrete structures based on visual inspection information. ACI Struct. J..

[22] Liu W., Hunsperger R.G., Chajes M.J., Folliard K.J., Kunz E. (2002). Corrosion Detection of Steel Cables using Time Domain Reflectometry. J. Mater. Civ. Eng..

[23] Li W., Xu C., Ho S.C.M., Wang B., Song G. (2017). Monitoring concrete deterioration due to reinforcement corrosion by integrating acoustic emission and FBG strain measurements. Sensors.

[24] Sharma S., Mukherjee A. (2015). Ultrasonic guided waves for monitoring corrosion in submerged plates. Struct. Control Health Monit..

[25] Yadav I.N., Thapa K.B. (2020). Fatigue damage model of concrete materials. Theor. Appl. Fract. Mech..

[26] Lü P., Li Q., Song Y. (2004). Damage constitutive of concrete under uniaxial alternate tension-compression fatigue loading based on double bounding surfaces. Int. J. Solids Struct..

[27] Yao Y., Glisic B. (2015). Detection of steel fatigue cracks with strain sensing sheets based on large area electronics. Sensors.

[28] Yaich A., El Hami A. (2019). Multiaxial fatigue damage estimation of structures under random vibrations using Matsubara’s criterion. Int. J. Fatigue.

[29] Blunt D.M., Keller J.A. (2006). Detection of a fatigue crack in a UH-60A planet gear carrier using analysis. Mech. Syst. Signal Process..

[30] Gao J.X., Heng F., Yuan Y.P., Liu Y.Y. (2023). Fatigue reliability analysis of composite material considering the growth of effective stress and critical stiffness. Aerospace.

[31] Cheng H.C., Hwu F.S. (2008). Fatigue reliability analysis of composites based on residual strength. Adv. Compos. Mater..

[32] Noorsuhada M. (2016). An overview on fatigue damage assessment of reinforced concrete structures with the aid of acoustic emission technique. Constr. Build. Mater..

[33] Aymerich F., Staszewski W.J. (2010). Impact damage detection in composite laminates using nonlinear acoustics. Compos. A Appl. Sci. Manuf..

[34] Saeedifar M., Mansvelder J., Mohammadi R., Zarouchas D. (2019). Using Passive and Active Acoustic Methods for Impact Damage Assessment of Composite Structures. Compos. Struct..

[35] Xia Q., Li H., Lu A., Tian Q., Liu J. (2017). Damage analysis of concrete members containing expansive agent by mechanical and acoustic methods. Eng. Fail. Anal..

[36] Papa I., Lopresto V., Langella A. (2021). Ultrasonic inspection of composites materials: Application to detect impact damage. Int. J. Lightweight Mater. Manuf..

[37] Mac V.H., Huh J., Doan N.S., Shin G., Lee B.Y. (2020). Thermography-Based Deterioration Detection in Concrete Bridge Girders Strengthened with Carbon Fiber-Reinforced Polymer. Sensors.

[38] Chen G., Mu H., Pommerenke D., Drewniak J.L. (2004). Damage detection of reinforced concrete beams with novel distributed crack/strain sensors. Struct. Health Monit..

[39] Li J., Hao H., Fan K., Brownjohn J. (2015). Development and application of a relative displacement sensor for structural health monitoring of composite bridges. Struct. Control Health Monit..

[40] Chu X., Zhou Z., Deng G., Duan X., Jiang X. (2019). An Overall Deformation Monitoring Method of Structure Based on Tracking Deformation Contour. Appl. Sci..

[41] D’Orazio T., Leo M., Distante A., Guaragnella C., Pianese V., Cavaccini G. (2008). Automatic ultrasonic inspection for internal defect detection in composite materials. NDT E Int..

[42] Ma F. (2021). Recent Advances in the GPR Detection of Grouting Defects behind Shield Tunnel Segments. Remote Sens..

[43] Lei W., Hou F., Xi J., Tan Q., Xu M., Jiang X., Liu Q., Gu Q. (2019). Automatic hyperbola detection and fitting in GPR B-scan image. Autom. Constr..

[44] Li S., Zhao X., Zhou G. (2019). Automatic pixel-level multiple damage detection of concrete structure using fully convolutional network. Comput.-Aided Civ. Infrastruct. Eng..

[45] Simoncelli M., Aloisio A., Zucca M., Venturi G., Alaggio R. (2023). Intensity and location of corrosion on the reliability of a steel bridge. J. Constr. Steel Res..

[46] Wan H., Gao L., Yuan Z., Qu H., Sun Q., Cheng H., Wang R. (2023). A novel transformer model for surface damage detection and cognition of concrete bridges. Expert Syst. Appl..

[47] Zhang C., Chang C.C., Jamshidi M. (2019). Concrete bridge surface damage detection using a single-stage detector. Comput. Aided Civ. Infrastruct. Eng..

[48] Zhao S., Kang F., Li J., Ma C. (2021). Structural health monitoring and inspection of dams based on UAV photogrammetry with image 3D reconstruction. Autom. Constr..

[49] Reagan D., Sabato A., Niezrecki C. (2018). Feasibility of using digital image correlation for unmanned aerial vehicle structural health monitoring of bridges. Struct. Health Monit..

[50] Ding W., Yang H., Yu K., Shu J. (2023). Crack detection and quantification for concrete structures using UAV and transformer. Autom. Constr..

[51] Zhao S., Kang F., Li J. (2022). Concrete dam damage detection and localisation based on YOLOv5s-HSC and photogrammetric 3D reconstruction. Autom. Constr..

[52] Zhu Y., Tang H. (2023). Automatic damage detection and diagnosis for hydraulic structures using drones and artificial intelligence techniques. Remote Sens..

[53] Silva L.A., Leithardt V.R.Q., Batista V.F.L., González G.V., Santana J.F.D.P. (2023). Automated Road Damage Detection using UAV Images and Deep Learning Techniques. IEEE Access.

[54] Kim H., Narazaki Y., Spencer B.F. (2023). Automated bridge component recognition using close-range images from unmanned aerial vehicles. Eng. Struct..

[55] Amieghemen G.E., Sherif M.M. (2023). Deep convolutional neural network ensemble for pavement crack detection using high elevation UAV images. Struct. Infrastruct. Eng..

[56] Ali R., Kang D., Suh G., Cha Y.J. (2021). Real-time multiple damage mapping using autonomous UAV and deep faster region-based neural networks for GPS-denied structures. Autom. Constr..

[57] Hellstein P., Szwedo M. (2016). 3D thermography in non-destructive testing of composite structures. Meas. Sci. Technol..

[58] Zhang R., Li H., Duan K., You S., Liu K., Wang F., Hu Y. (2020). Automatic detection of earthquake-damaged buildings by integrating UAV oblique photography and infrared thermal imaging. Remote Sens..

[59] Omar T., Nehdi M.L. (2017). Remote sensing of concrete bridge decks using unmanned aerial vehicle infrared thermography. Autom. Constr..

[60] Žnidarič A., Kreslin M., Anžlin A., Krivic A. (2020). Detection of Delaminated and Cracked Concrete with Unmanned Aerial Vehicles. Routes/Roads.

[61] Deane S., Avdelidis N.P., Ibarra-Castanedo C., Williamson A.A., Withers S., Zolotas A., Maldague X.P.V., Ahmadi M., Pant S., Genest M. (2023). Development of a thermal excitation source used in an active thermographic UAV platform. Quant. Infrared Thermogr. J..

[62] Aldave I.J., Bosom P.V., González L.V., De Santiago I.L., Vollheim B., Krausz L., Georges M. (2013). Review of thermal imaging systems in composite defect detection. Infrared Phys. Technol..

[63] Alexander Q.G., Hoskere V., Narazaki Y., Maxwell A., Spencer B.F. (2022). Fusion of thermal and RGB images for automated deep learning based crack detection in civil infrastructure. AI Civ. Eng..

[64] Yan Y., Mao Z., Wu J., Padir T., Hajjar J.F. (2021). Towards automated detection and quantification of concrete cracks using integrated images and lidar data from unmanned aerial vehicles. Struct. Contr. Health Monit..

[65] Erkal B.G., Hajjar J.F. (2017). Laser-based surface damage detection and quantification using predicted surface properties. Autom. Constr..

[66] Park H.S., Lee H.M., Adeli H., Lee I. (2007). A new approach for health monitoring of structures: Terrestrial laser scanning. Comput.-Aided Civ. Inf..

[67] Bolourian N., Hammad A. (2020). LiDAR-equipped UAV path planning considering potential locations of defects for bridge inspection. Autom. Constr..

[68] Chen S.E., Liu W., Bian H., Smith B. (2013). 3D LiDAR scans for bridge damage evaluations. Forensic Engineering 2012: Gateway to a Safer Tomorrow.

[69] Valença J., Puente I., Júlio E.N.B.S., González-Jorge H., Arias-Sánchez P. (2017). Assessment of cracks on concrete bridges using image processing supported by laser scanning survey. Constr. Build. Mater..

[70] Bolourian N., Soltani M.M., Albahria A.H., Hammad A. High level framework for bridge inspection using LiDAR-equipped UAV. Proceedings of the ISARC—International Symposium on Automation and Robotics in Construction.

[71] Kaartinen E., Dunphy K., Sadhu A. (2022). LiDAR-based structural health monitoring: Applications in civil infrastructure systems. Sensors.

[72] Farahani B.V., Barros F., Sousa P.J., Cacciari P.P., Tavares P.J., Futai M.M., Moreira P. (2019). A coupled 3D laser scanning and digital image correlation system for geometry acquisition and deformation monitoring of a railway tunnel. Tunn. Undergr. Space.

[73] Araújo G.R.M.B.D., Corsi A.C., Macedo E.S.D., Futai M.M. (2023). Application of digital technologies in landslide prediction, mapping, and monitoring. Soils Rocks.

[74] Wang Q., Zhao P., Lv Y., Qin L. (2023). Monitoring Corrosion of Steel Bar in Concrete Using Ultrasonic Wave. Int. J. Adv. Eng. Technol..

[75] Shetty S., Banerjee S., Tallur S., Desai Y.M. (2023). Real-time monitoring of residual strength in corroding steel reinforcement using ultrasonic-guided waves and multi-physics modelling. J. Adhes. Sci. Technol..

[76] Rodriguez S., Gayoux V., Ducasse E., Castaings M., Patteeuw N. (2023). Ultrasonic imaging of buried defects in rails. NDT E Int..

[77] Gharehdash S., Laleh M., Sainsbury D., Barzegar M., Sainsbury B.A. (2023). Low-Frequency ultrasonic tomography of Corrosion-induced damage patterns on naturally corroded solid reinforcing bar rock bolts. Constr. Build. Mater..

[78] Khademi P., Mousavi M., Dackermann U., Gandomi A.H. (2023). Time–frequency analysis of ultrasonic signals for quality assessment of bonded concrete. Constr. Build. Mater..

[79] Zhan H., Jiang H., Liang Z., Jiang R. (2023). Nondestructive In Situ Imaging of Preexisting Cracks in a Concrete Bridge Using Ultrasonic Coda Wave. J. Struct. Eng..

[80] Ge L., Li Q., Wang Z., Li Q., Lu C., Dong D., Wang H. (2023). High-resolution ultrasonic imaging technology for the damage of concrete structures based on total focusing method. Comput. Electr. Eng..

[81] Bagheri A., Rizzo P., Li K. (2017). Ultrasonic imaging algorithm for the health monitoring of pipes. J. Civ. Struct. Health Monit..

[82] Xu J., Wei H. (2019). Ultrasonic testing analysis of concrete structure based on S transform. Shock. Vib..

[83] Dinh K., Gucunski N., Tran K., Novo A., Nguyen T. (2021). Full-resolution 3D imaging for concrete structures with dual-polarization GPR. Autom. Constr..

[84] Bertolini L., D’Amico F., Napolitano A., Bianchini Ciampoli L., Gagliardi V., Romer Diezmos Manalo J. (2023). A BIM-Based Approach for Pavement Monitoring Integrating Data from Non-Destructive Testing Methods (NDTs). Infrastructures.

[85] Salari A., Esposito G., Catapano I., Soldovieri F., Erricolo D. (2023). A Compact and Light-Weight Ground Penetrating Radar System for Unmanned Aerial Vehicles. Proceedings of the 2023 United States National Committee of URSI National Radio Science Meeting (USNC-URSI NRSM).

[86] Morris I., Abdel-Jaber H., Glisic B. (2019). Quantitative attribute analyses with ground penetrating radar for infrastructure assessments and structural health monitoring. Sensors.

[87] Morris I.M., Kumar V., Glisic B. (2021). Predicting material properties of concrete from ground-penetrating radar attributes. Struct. Health Monit..

[88] Xiang Z., Ou G., Rashidi A. (2020). Integrated approach to simultaneously determine 3D location and size of rebar in GPR data. J. Perform. Constr. Facil..

[89] Ghodoosi F., Bagchi A., Zayed T., Hosseini M.R. (2018). Method for developing and updating deterioration models for concrete bridge decks using GPR data. Autom. Constr..

[90] Kumar V., Morris I.M., Lopez S.A., Glisic B. (2021). Identifying spatial and temporal variations in concrete bridges with ground penetrating radar attributes. Remote Sens..

[91] Gagliardi V., Tosti F., Bianchini Ciampoli L., Battagliere M.L., D’Amato L., Alani A.M., Benedetto A. (2023). Satellite remote sensing and non-destructive testing methods for transport infrastructure monitoring: Advances, challenges and perspectives. Remote Sens..

[92] Talledo D.A., Miano A., Bonano M., Di Carlo F., Lanari R., Manunta M., Meda A., Mele A., Prota A., Saetta A. (2022). Satellite radar interferometry: Potential and limitations for structural assessment and monitoring. J. Build. Eng..

[93] Shen N., Chen L., Liu J., Wang L., Tao T., Wu D., Chen R. (2019). A review of global navigation satellite system (GNSS)-based dynamic monitoring technologies for structural health monitoring. Remote Sens..

[94] Hoppe E.J., Novali F., Rucci A., Fumagalli A., Del Conte S., Falorni G., Toro N. (2019). Deformation monitoring of posttensioned bridges using high-resolution satellite remote sensing. J. Bridge Eng..

[95] Mazzanti P., Perissin D., Rocca A. Structural health monitoring of dams by advanced satellite SAR interferometry: Investigation of past processes and future monitoring perspectives. Proceedings of the 7th Internation Conference on Structural Health Monitoring of Intelligent Infrastructure.

[96] Bakon M., Perissin D., Lazecky M., Papco J. (2014). Infrastructure non-linear deformation monitoring via satellite radar interferometry. Proc. Technol..

[97] Entezami A., De Michele C., Arslan A.N., Behkamal B. (2022). Detection of partially structural collapse using long-term small displacement data from satellite images. Sensors.

[98] Wang H., Chang L., Markine V. (2018). Structural health monitoring of railway transition zones using satellite radar data. Sensors.

[99] Tonelli D., Caspani V.F., Valentini A., Rocca A., Torboli R., Vitti A., Perissin D., Zonta D. (2023). Interpretation of Bridge Health Monitoring Data from Satellite InSAR Technology. Remote Sens..

[100] Caprino A., Puliero S., Lorenzoni F., Floris M., da Porto F. (2023). Satellite SAR Interferometry and On-Site Traditional SHM to Monitor the Post-Earthquake Behavior of the Civic Tower in L’Aquila (Abruzzo Region, Italy). Remote Sens..

[101] Ziou D., Tabbone S. (1998). Edge detection techniques-an overview. Pattern Recognit. Image Anal..

[102] Dharampal V.M. (2015). Methods of image edge detection: A review. J. Electr. Electron. Syst..

[103] Maini R., Aggarwal H. (2009). Study and comparison of various image edge detection techniques. Int. J. Image Process. IJIP.

[104] Hoang N.D., Nguyen Q.L. (2018). Metaheuristic optimized edge detection for recognition of concrete wall cracks: A comparative study on the performances of roberts, prewitt, canny, and sobel algorithms. Adv. Civ. Eng..

[105] Prasetyo A., Yuniarto E.M., Suprobo P., Tambusay A. (2022). Application of Edge Detection Technique for Concrete Surface Crack Detection. Proceedings of the 2022 International Seminar on Intelligent Technology and Its Applications (ISITIA).

[106] Han H., Deng H., Dong Q., Gu X., Zhang T., Wang Y. (2021). An advanced Otsu method integrated with edge detection and decision tree for crack detection in highway transportation infrastructure. Adv. Mater. Sci..

[107] Jena K.K., Mishra S., Mishra S., Bhoi S.K. (2019). Unmanned aerial vehicle assisted bridge crack severity inspection using edge detection methods. Proceedings of the 2019 Third International conference on I-SMAC (IoT in Social, Mobile, Analytics and Cloud) (I-SMAC).

[108] Nguyen H.N., Kam T.Y., Cheng P.Y. (2014). An automatic approach for accurate edge detection of concrete crack utilizing 2D geometric features of crack. J. Signal Process. Syst..

[109] Abid Hasan S.M., Ko K. (2016). Depth edge detection by image-based smoothing and morphological operations. J. Comput. Des. Eng..

[110] Nguyen A., Gharehbaghi V., Le N.T., Sterling L., Chaudhry U.I., Crawford S. (2023). ASR crack identification in bridges using deep learning and texture analysis. Structures.

[111] Hoang N.D., Huynh T.C., Tran V.D. (2021). Concrete spalling severity classification using image texture analysis and a novel jellyfish search optimized machine learning approach. Adv. Civ. Eng..

[112] O’Byrne M., Schoefs F., Ghosh B., Pakrashi V. (2013). Texture analysis based damage detection of ageing infrastructural elements. Comput.-Aided Civ. Inf..

[113] O’Byrne M., Ghosh B., Pakrashi V., Schoefs F. Texture analysis based detection and classification of surface features on ageing infrastructure elements. Proceedings of the Bridge and Concrete Research in Ireland Conference (BCRI 2012).

[114] Bu G.P., Chanda S., Guan H., Jo J., Blumenstein M., Loo Y.C. (2015). Crack detection using a texture analysis-based technique for visual bridge inspection. Electron. J. Struct. Eng..

[115] Hoang N.D., Nguyen Q.L. (2020). A novel approach for automatic detection of concrete surface voids using image texture analysis and history-based adaptive differential evolution optimized support vector machine. Adv. Civ. Eng..

[116] Nooralishahi P., Ramos G., Pozzer S., Ibarra-Castanedo C., Lopez F., Maldague X.P. (2022). Texture analysis to enhance drone-based multi-modal inspection of structures. Drones.

[117] Zhu L., Dang F., Xue Y., Ding W., Zhang L. (2019). Analysis of micro-structural damage evolution of concrete through coupled X-ray computed tomography and gray-level co-occurrence matrices method. Constr. Build. Mater..

[118] He J., Shao L., Li Y., Wang K., Liu W. (2023). Pavement damage identification and evaluation in UAV-captured images using gray level co-occurrence matrix and cloud model. J. King Saud. Univ. Comput. Inf..

[119] Brown L.G. (1992). A survey of image registration techniques. CM Comput. Surv..

[120] Hsu W.Y. (2015). A novel image registration algorithm for indoor and built environment applications. Comput.-Aided Civ. Inf..

[121] Park J., Cai H., Perissin D. (2018). Bringing information to the field: Automated photo registration and 4D BIM. J. Comput. Civ. Eng..

[122] Li H., Ding W., Cao X., Liu C. (2017). Image registration and fusion of visible and infrared integrated camera for medium-altitude unmanned aerial vehicle remote sensing. Remote Sens..

[123] Quan D., Wang S., Gu Y., Lei R., Yang B., Wei S., Hou B., Jiao L. (2022). Deep feature correlation learning for multi-modal remote sensing image registration. IEEE Trans. Geosci. Remote Sens..

[124] Vishwakarma H., Katiyar S.K. (2018). Accuracy assessment of projective transformation based hybrid approach for automatic satellite image registration. Int. J. Civ. Eng. Technol..

[125] Jianchao Y., Chern C.T., Hwang Y.C., Adrian Y.C. (2001). Automatic satellite image registration based on intensity matching. Proceedings of the IGARSS 2001, Scanning the Present and Resolving the Future, International Geoscience and Remote Sensing Symposium (Cat. No. 01CH37217).

[126] Shang Z., Shen Z. (2018). Multi-point vibration measurement and mode magnification of civil structures using video-based motion processing. Autom. Constr..

[127] Li H.Y., Huang C.Y., Wang C.Y. (2023). Measurement of Cracks in Concrete Bridges by Using Unmanned Aerial Vehicles and Image Registration. Drones.

[128] Foryś P., Sitnik R., Markiewicz J., Bunsch E. (2023). Fast adaptive multimodal feature registration (FAMFR): An effective high-resolution point clouds registration workflow for cultural heritage interiors. Herit. Sci..

[129] Kong X., Li J. (2019). Non-contact fatigue crack detection in civil infrastructure through image overlapping and crack breathing sensing. Autom. Constr..

[130] Zhang Y., Cao G., Wang G. (2023). Vision-based measurement for the transverse-longitudinal-rotational displacement of hoisting rope by modified Lucas-Kanade algorithm. IEEE Trans. Instrum. Meas..

[131] Yang Y., Yu X.B. (2016). Real time measurement of the dynamic displacement field of a large-scale arch-truss bridge by remote sensing technology. Proceedings of the 2016 IEEE SENSORS.

[132] Guo J. (2016). Dynamic displacement measurement of large-scale structures based on the Lucas–Kanade template tracking algorithm. Mech. Syst. Signal Process..

[133] Daubechies I., Defrise M., De Mol C. (2004). An iterative thresholding algorithm for linear inverse problems with a sparsity constraint. Commun. Pure Appl. Math..

[134] Lei M., Liu L., Shi C., Tan Y., Lin Y., Wang W. (2021). A novel tunnel-lining crack recognition system based on digital image technology. Tunn. Undergr. Space Technol..

[135] Akagic A., Buza E., Omanovic S., Karabegovic A. (2018). Pavement crack detection using Otsu thresholding for image segmentation. Proceedings of the 2018 41st International Convention on Information and Communication Technology, Electronics and Microelectronics (MIPRO).

[136] Singh P., Shree R. (2020). A new homomorphic and method noise thresholding based despeckling of SAR image using anisotropic diffusion. J. King Saud. Univ. Comput. Inf..

[137] Bao P., Zhang L., Wu X. (2005). Canny edge detection enhancement by scale multiplication. IEEE Trans. Pattern Anal. Mach. Intell..

[138] Nnolim U.A. (2020). Automated crack segmentation via saturation channel thresholding, area classification and fusion of modified level set segmentation with Canny edge detection. Heliyon.

[139] Nhat-Duc H., Nguyen Q.L., Tran V.D. (2018). Automatic recognition of asphalt pavement cracks using metaheuristic optimized edge detection algorithms and convolution neural network. Autom. Constr..

[140] Wang G., Peter W.T., Yuan M. (2018). Automatic internal crack detection from a sequence of infrared images with a triple-threshold Canny edge detector. Meas. Sci. Technol..

[141] Yeom J., Jung M., Kim Y. (2017). Detecting damaged building parts in earthquake-damaged areas using differential seeded region growing. Int. J. Remote Sens..

[142] Asmussen P., Conrad O., Günther A., Kirsch M., Riller U. (2015). Semi-automatic segmentation of petrographic thin section images using a “seeded-region growing algorithm” with an application to characterize wheathered subarkose sandstone. Comput. Geosci..

[143] Guo Q., Wang Y., Yang S., Xiang Z. (2022). A method of blasted rock image segmentation based on improved watershed algorithm. Sci. Rep..

[144] Jagadeesh A., Ong G.P., Su Y.M. Evaluation of pervious concrete pore network properties using watershed segmentation approach. Proceedings of the International Airfield and Highway Pavements Conference 2019.

[145] Sahari Moghaddam A., Rezazadeh Azar E., Mejias Y., Bell H. (2020). Estimating stripping of asphalt coating using k-means clustering and machine learning–based classification. J. Comput. Civ. Eng..

[146] Brilakis I.K., Soibelman L., Shinagawa Y. (2006). Construction site image retrieval based on material cluster recognition. Adv. Eng. Inform..

[147] Alamdari M.M., Rakotoarivelo T., Khoa N.L.D. (2017). A spectral-based clustering for structural health monitoring of the Sydney Harbour Bridge. Mech. Syst. Signal Process..

[148] Ye X.W., Jin T., Yun C.B. (2019). A review on deep learning-based structural health monitoring of civil infrastructures. Smart Struct. Syst..

[149] Sony S., Dunphy K., Sadhu A., Capretz M. (2021). A systematic review of convolutional neural network-based structural condition assessment techniques. Eng. Struct..

[150] Resende L., Finotti R., Barbosa F., Garrido H., Cury A., Domizio M. (2023). Damage identification using convolutional neural networks from instantaneous displacement measurements via image processing. Struct. Health Monit..

[151] Mantawy I.M., Mantawy M.O. (2022). Convolutional neural network based structural health monitoring for rocking bridge system by encoding time-series into images. Struct. Control Health Monit..

[152] Avci O., Abdeljaber O., Kiranyaz S., Hussein M., Gabbouj M., Inman D.J. (2021). A review of vibration-based damage detection in civil structures: From traditional methods to Machine Learning and Deep Learning applications. Mech. Syst. Signal Process..

[153] Wang Z., Cha Y.J. (2022). Unsupervised machine and deep learning methods for structural damage detection: A comparative study. Eng. Rep..

[154] Salunkhe A.A., Gobinath R., Vinay S., Joseph L. (2022). Progress and trends in image processing applications in civil engineering: Opportunities and challenges. Adv. Civ. Eng..

[155] Flah M., Nunez I., Ben Chaabene W., Nehdi M.L. (2021). Machine learning algorithms in civil structural health monitoring: A systematic review. Arch. Comput. Methods Eng..

[156] Bao Y., Li H. (2021). Machine learning paradigm for structural health monitoring. Struct. Health Monit..

[157] Dong C.Z., Catbas F.N. (2021). A review of computer vision–based structural health monitoring at local and global levels. Struct. Health Monit..

[158] Kim H., Ahn E., Shin M., Sim S.H. (2019). Crack and noncrack classification from concrete surface images using machine learning. Struct. Health Monit..

[159] Valikhani A., Jaberi Jahromi A., Pouyanfar S., Mantawy I.M., Azizinamini A. (2021). Machine learning and image processing approaches for estimating concrete surface roughness using basic cameras. Comput.-Aided Civ. Inf..

[160] Rafiei M.H., Adeli H. (2017). A novel machine learning-based algorithm to detect damage in high-rise building structures. Struct. Des. Tall Spec. Build..

[161] Tang Z., Guo L., Zheng T., Li Z., Sun R., Huang K. (2022). A combined machine learning and numerical approach for evaluating the uncertainty of 3D angle-interlock woven composites. Compos. Struct..

[162] Silva M., Santos A., Santos R., Figueiredo E., Sales C., Costa J.C. (2019). Deep principal component analysis: An enhanced approach for structural damage identification. Struct. Health Monit..

[163] Kaloni S., Singh G., Tiwari P. (2021). Nonparametric damage detection and localization model of framed civil structure based on local gravitation clustering analysis. J. Build. Eng..

[164] Coraça E.M., Ferreira J.V., Nóbrega E.G. (2023). An unsupervised structural health monitoring framework based on Variational Autoencoders and Hidden Markov Models. Reliab. Eng. Syst. Saf..

[165] Eltouny K., Gomaa M., Liang X. (2023). Unsupervised Learning Methods for Data-Driven Vibration-Based Structural Health Monitoring: A Review. Sensors.

[166] Wang Z., Cha Y.J. (2021). Unsupervised deep learning approach using a deep auto-encoder with a one-class support vector machine to detect damage. Struct. Health Monit..

[167] Liu J., Li K. (2022). Research on an Improved SOM Model for Damage Identification of Concrete Structures. Appl. Sci..

[168] Kohiyama M., Oka K., Yamashita T. (2020). Detection method of unlearned pattern using support vector machine in damage classification based on deep neural network. Struct. Control Health Monit..

[169] Chen J., Liu D. (2021). Bottom-up image detection of water channel slope damages based on superpixel segmentation and support vector machine. Adv. Eng. Inform..

[170] Sun L., Shang Z., Xia Y., Bhowmick S., Nagarajaiah S. (2020). Review of bridge structural health monitoring aided by big data and artificial intelligence: From condition assessment to damage detection. J. Struct. Eng..

[171] Barkhordari M.S., Barkhordari M.M., Armaghani D.J., Rashid A.S.A., Ulrikh D.V. (2022). Hybrid Wavelet Scattering Network-Based Model for Failure Identification of Reinforced Concrete Members. Sustainability.

